# Fighting MDR-*Klebsiella pneumoniae* Infections by a Combined Host- and Pathogen-Directed Therapeutic Approach

**DOI:** 10.3389/fimmu.2022.835417

**Published:** 2022-02-14

**Authors:** Noemi Poerio, Tommaso Olimpieri, Lucia Henrici De Angelis, Federica De Santis, Maria Cristina Thaller, Marco Maria D’Andrea, Maurizio Fraziano

**Affiliations:** ^1^ Department of Biology, University of Rome “Tor Vergata”, Rome, Italy; ^2^ Department of Medical Biotechnologies, University of Siena, Siena, Italy

**Keywords:** liposomes, phosphatidylinositol 5-phospate, host-directed therapy, bacteriophages, phage therapy, MDR, *Klebsiella pneumoniae*

## Abstract

*Klebsiella pneumoniae* is an opportunistic pathogen that is very difficult to treat mainly due to its high propensity to acquire complex resistance traits. Notably, multidrug resistance (MDR)-*Klebsiella pneumoniae* (KP) infections are responsible for 22%–72% of mortality among hospitalized and immunocompromised patients. Although treatments with new drugs or with combined antibiotic therapies have some degree of success, there is still the urgency to investigate and develop an efficient approach against MDR-KP infections. In this study, we have evaluated, in an *in vitro* model of human macrophages, the efficacy of a combined treatment consisting of apoptotic body-like liposomes loaded with phosphatidylinositol 5-phosphate (ABL/PI5P) and φBO1E, a lytic phage specific for the major high-risk clone of KPC-positive MDR-KP. Results show that ABL/PI5P did not affect in a direct manner KKBO-1 viability, being able to reduce only the intracellular KKBO-1 bacterial load. As expected, φBO1E was effective mainly on reducing extracellular bacilli. Importantly, the combination of both treatments resulted in a simultaneous reduction of both intracellular and extracellular bacilli. Moreover, the combined treatment of KKBO-1-infected cells reduced proinflammatory TNF-α and IL-1β cytokines and increased anti-inflammatory TGF-β cytokine production. Overall, our data support the therapeutic value of a combined host- and pathogen-directed therapy as a promising approach, alternative to single treatments, to simultaneously target intracellular and extracellular pathogens and improve the clinical management of patients infected with MDR pathogens such as MDR-KP.

## Introduction

*Klebsiella pneumoniae* (KP) is a major Gram-negative opportunistic bacterium included in the list of ESKAPE pathogens (*Enterococcus faecium, Staphylococcus aureus, Klebsiella pneumoniae, Acinetobacter baumannii, Pseudomonas aeruginosa, Enterobacter spp.*), known to cause opportunistic infections and characterized by a remarkable antibiotic resistance level due to accumulation of mobile antimicrobial resistance genes gained by horizontal gene transfer (1). Notably, multidrug-resistant (MDR)-KP infections, such as those sustained by *K. pneumoniae* carbapenemase (KPC)-producing *K. pneumoniae*, are associated with high morbidity and mortality rates, which can vary from 22% to 72% (2) and affect in particular hospitalized and immunocompromised patients. Although combined antibiotic therapies have been the most used in the last years, they have not given often satisfying results, opening the urgency to investigate and develop an efficient approach to treat MDR-KP infections (3).

We already demonstrated that apoptotic body-like (ABL) liposomes composed by a phosphatidylserine (PS) outer leaflet and an inner leaflet consisting of a bioactive lipid involved in phagocytosis process, are able to enhance macrophage intracellular bacterial killing irrespective of their drug resistance and species (4, 5). Phagocytosis is a pivotal effector mechanism of innate immunity, and second lipid messengers play a key role in phagosome formation and maturation, recruiting signal protein by means of specific lipid-binding domain, making possible the development of phagolysosome, the effective microbicidal compartment (6). In particular, the bioactive lipid phosphatidylinositol 5-phoshate (PI5P) is involved in the late stages of phagosome maturation, taking part in the vesicular traffic and in noncanonical autophagy process (6, 7). Moreover, *K. pneumoniae* is known to be able to interfere with phagocytosis process by carrying out intracellular survival strategies interfering with phagolysosome generation, achieving survival within vacuole compartment (*Klebsiella*-containing vacuole (KCV)) and avoiding their delivery to lysosomes (8). Being an extracellular-capsulated bacterium, *K. pneumoniae* escapes macrophage phagocytosis and thus intracellular internalization. Thanks to this ability, *K. pneumoniae* is also able to form biofilms, which play an important role in interfering with the immune response, by both avoiding opsonization mechanisms and by modulating inflammatory response, contributing to the development of invasive and chronic opportunistic infections (9). On this scenario, the usage of bacteriophages for microbicide therapeutic purpose, also known as phage therapy, could represent an interesting and attractive option, as bacteriophages are (i) highly abundant in the environment, (ii) usually do not present any significant adverse safety concerns, (iii) are able to specifically proliferate within bacterial targets, without interfering with the whole host microbiota, and (iv) may have a potential role in the disruption of biofilms (10, 11). On these grounds, we propose a combined host- and pathogen-directed treatment, composed of (i) ABL carrying the second lipid messenger PI5P and (ii) a phage (φBO1E) specific against KPC-positive KP (KPC-KP) clinical strains belonging to the Sequence Type (ST) 258 clade II, aimed to simultaneously target intracellular and extracellular bacilli.

## Method

### Cell Line

Human promonocytic THP-1 leukemia cell line was supplied by the European Collection of Cell Culture, grown in RPMI-1640 containing fetal bovine serum (10%), gentamycin (5 μg/ml), l-glutammine (2 mM), nonessential amino acids (1 mM), sodium pyruvate (1 mM), and cultured in 75 cm^2^ polystyrene flasks. Before experiments, cells (2.5 × 10^5^ per well) were seeded in 48-well plates and were then induced to differentiate (dTHP-1) by stimulation for 72 h with phorbol 12-myristate 13-acetate (PMA) (20 ng/ml) and used as a model of human macrophages.

### Liposome

Apoptotic body-like liposomes (ABLs) carrying 1,2-dioleoyl-sn-glycero-3-phospho (1′-myo-inositol-5′-phosphate) (PI5P, Avanti Polar Lipids) (ABL/PI5P) were produced and quantified as previously described (5).

### Bacteria

The *K. pneumoniae* KKBO-1 clinical strain was used as a representative of the major clonal lineage of KPC-KP diffused at a worldwide scale. Single colonies of KKBO-1 were collected by streaking on Trypticase soy agar (TSA) (BD Difco™, BD Bioscience, Franklin Lakes, NJ, USA) and then suspended in 15 ml of Trypticase soy broth (TSB) (BD Difco™). Bacteria were grown in Erlenmeyer flasks at 37°C under stirring for 18 h, and their growth was monitored by measuring the optical density at the wavelength of 600 nm by Varioskan LUX Multimode Microplate Reader (Thermo Fisher Scientific, Waltham, MA, USA). Bacteria were stored at −80°C until use after suspension in Microorganism Preservation System-Protect (Technical Service Consultants Ltd., Heywood, UK).

### Bacteriophage

The φBO1E bacteriophage, a lytic phage of the *Autographiviridae* family able to selectively kill *K. pneumoniae* strains belonging to the ST258 clade II, was isolated and characterized as described by D’Andrea et al. (12)

### Evaluation of *In Vitro* Bacterial Growth

To assess both extracellular and intracellular bacterial growth, dTHP-1 were plated at a concentration of 2.5 × 10^5^ cells/well and were infected with KKBO-1 for 1 h at 37°C at a multiplicity of infection (MOI) of 5, in the absence of gentamycin. Cells were then washed and incubated with ABL/PI5P (ABL:cell ratio 1:1) and/or φBO1E (KKBO-1:phage ratio 2:1) for further 2 h. In order to evaluate extracellular and intracellular bacterial growth, supernatants were collected, and macrophages lysed with deoxycholate 1% (Sigma Aldrich, St. Louis, MO, USA), respectively. Finally, samples were diluted in PBS-Tween 80 and CFU quantified by plating bacilli in triplicate on TSA.

### Enzyme-Linked Immunosorbent Assay

dTHP-1 were infected or not with KKBO-1 (MOI 5) for 1 h, and after cells were washed twice with PBS supplemented with gentamycin 500 µg/ml (Lonza), and one last time with PBS only in order to remove all extracellular bacilli. Cells were then stimulated or not for 18 h with ABL/PI5P (ABL:cell ratio 1:1) and/or φBO1E (KKBO-1: phage ratio 2:1). Thereafter, supernatants were collected and stored at −20°C until analysis. The levels of tumor necrosis factor-α (TNF-α), interleukin-1β (IL-1β), and transforming growth factor-beta (TGF-β) in the supernatants of dTHP-1 were measured by human TNF-α DuoSet^®^ ELISA Development Systems, human IL-1β DuoSet^®^ ELISA Development Systems and human TGF-β DuoSet^®^ ELISA Development Systems (all by R&D System, Minneapolis, MN, USA) according to the manufacturer’s instructions.

## Results and Discussion

Nowadays, infection diseases caused by MDR Gram-negative pathogens, such as KPC-KP, are one of the major global health concerns (3). Bacterial pathogens responsible for respiratory tract infections may cause pneumonia, which is a form of acute respiratory infection that affects the lungs and is caused by a number of infectious agents. In particular, opportunistic pathogens have been identified as causative agents of different respiratory tract infections and in the last years there was an increased incidence of MDR strains (5). In this context, infections due to MDR-KP are associated with a high mortality among hospitalized and immunocompromised patients, and the optimal antibiotic combination regimen to control and resolve such infections has not been established yet (3). On these grounds, the development of different strategies that may support standard pathogen-directed therapies (PDT), such as antibiotics, for the management of MDR-KP infections is considered urgent. It was already demonstrated that the contextual administration of host-directed therapies (HDTs), as immunomodulators, and antibiotics proved to be highly effective against MDR pathogens such as *Mycobacterium tuberculosis* and *P. aeruginosa* (13). Based on this evidence, we decided to combine an alternative PDT consisting in specific bacteriophages against KKBO-1 KPC-KP clinical strain (φBO1E) (12) with a novel HDT based on ABL/PI5P for the treatment of MDR-KP infections. In this context ABL/PI5P was chosen given its ability to enhance phagocytosis which represents one of the main innate immune response mechanisms that can be enhanced *via* HDT (14, 15).

We have recently demonstrated that ABL loaded with PI5P is able to enhance antimicrobial response in bronchoalveolar lavage (BAL) cells isolated from patients with pneumonia due to MDR pathogens, including MDR-KP (5). Furthermore, we reported that in an *in vivo* model of *Mycobacterium abscessus* chronic infection, the combined treatment with ABL/PI5P and amikacin promotes a significant additional reduction of both pulmonary mycobacterial burden and inflammatory response in comparison with single treatments, with no signs of kidney and liver toxicity (16).

**Figure 1:**
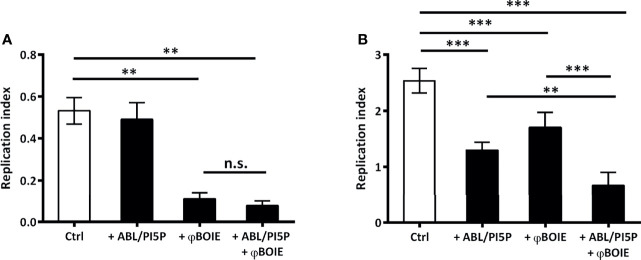
ABL/PI5P-φBO1E combined treatment reduces in an additive manner intracellular growth of KKBO-1. To assess both extracellular **(A)** and intracellular **(B)** bacterial growth, dTHP-1 were plated at the concentration of 2.5 × 10^5^ cells/well and were infected with KKBO-1 KPC-KP clinical strain, for 1 h at 37°C at a multiplicity of infection (MOI) of 5. Thereafter, extracellular bacilli were collected, adherent cells were lysed with 1% deoxycholate, and both were plated in triplicate on TSA (T0, immediately after 1 h of infection). After, cells were treated with ABL/PI5P and/or φBO1E (MOI 1:0.5 KPC-KP:phage) for further 2 h. Finally, extracellular bacilli were collected and cells were lysed with 1% deoxycholate, samples diluted in PBS-Tween 80 and CFU quantified by plating bacilli in triplicate on TSA. Replication index was calculated as the ratio between the CFU obtained 2 h after infection in the presence or absence of ABL/PI5P and/or and φBO1E the CFU obtained immediately after infection (T0), before the addition of the stimuli. The results are shown as mean ± standard deviation of the values obtained from triplicate of each condition. **p < 0.01, ***p < 0.001, and n.s. “not significant” by Student’s t-test.

Antibiotic administration represents the worldwide most common PDT used to treat infections so far, but, recently, alternative PDTs have been proposed, with phage therapy representing a very promising candidate to be used as supplement or even replacement of antibiotic treatments (17). This is particularly true considering that the pandemic dissemination of KPC-KP is mainly sustained by a single clonal lineage, i.e., strains belonging to the clade II of the clonal group 258. In this scenario, the use of bacteriophages is a promising approach, given the already existing evidence of their *in vivo* efficacy by using intranasal administration against a wide range of pathogens, comprising MDR-KP (18). Coherently with this evidence, we show that the treatment with ABL/PI5P is able to enhance the intracellular clearance (**Figure 1B**) of the KKBO-1 KPC-KP clinical strain, with no effect on bacilli viability (**Figure 1A**). Moreover, here, we demonstrated that φBO1E is able to kill extracellular KKBO-1 (**Figure 1A**), even though we noticed a reduction in intracellular microbial presence after phage treatment (**Figure 1B**). This may be due to the already described ability of some bacteriophages to penetrate inside macrophages or to the fortuitous engulfment of phage-infected bacteria by macrophages (19).

**Figure 2:**
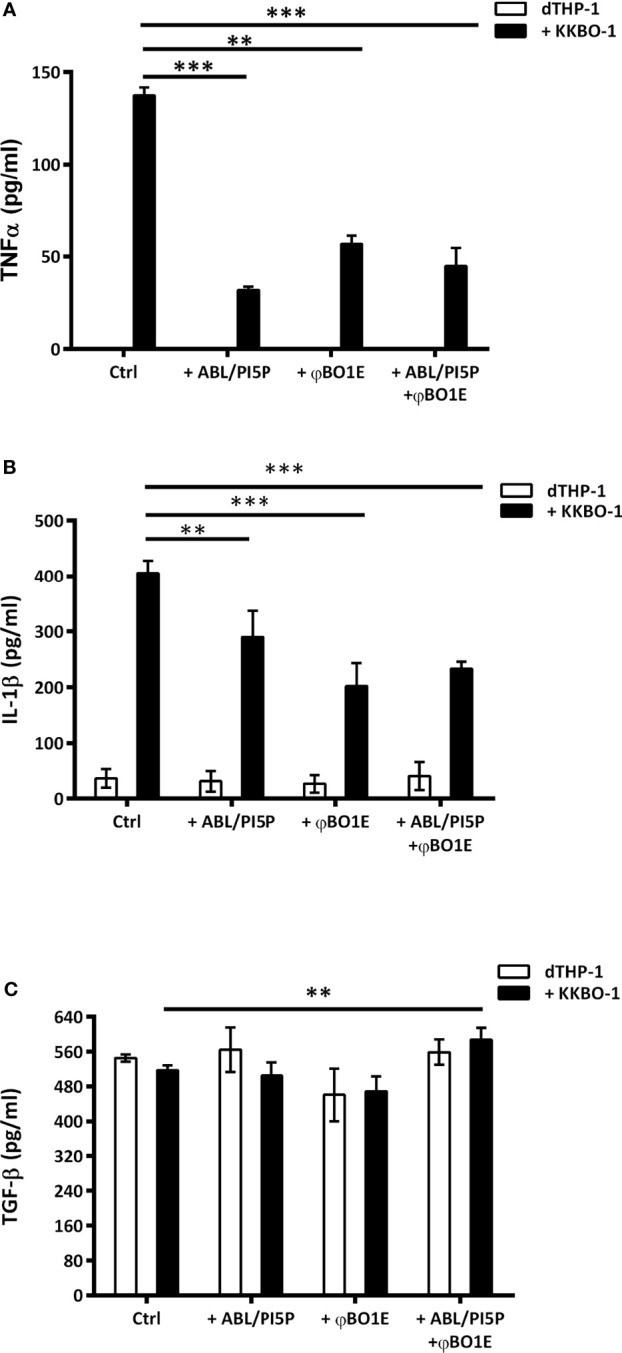
ABL/PI5P and/or φBO1E stimulation modulates cytokine production in KKBO-1-infected human macrophages. dTHP-1 cells were infected or not with KPC-KP KKBO-1 clinical strain and then stimulated or not with ABL/PI5P and/or φBO1E for 18 h. Thereafter supernatants were collected and stored at −20°C until analysis. The production of TNF-α **(A)**, IL-1β **(B)**, and TGF-β **(C)** was analyzed by ELISA. The results are shown as mean ± standard deviation of the values obtained from triplicate of each condition and are representative of three different experiments. ^**^ *p* < 0.01, ^***^ *p* < 0.001 by Student’s *t*-test.

More importantly, we also demonstrated that when combined, ABL/PI5P and φBO1E succeed in reducing extracellular bacterial burden and in enhancing antimicrobial activity of macrophages at once, producing an additive effect statistically more significant than the two single treatments (**Figure 1B**), without having any cytotoxic effect (**Supplementary Material S1**). Lastly, inflammation plays a crucial role as first-line defense mechanism in a context of infection, and a successful cytokine profile modulation is critical to design an effective HDT strategy. The net effect of an inflammatory response is determined by the balance between pro- and anti-inflammatory cytokines secreted by a broad range of cells, including macrophages, and is needed in order to avoid immunopathology (20). The ABL/PI5P ability in modulating cytokine production in a context of MDR pathogen infection has already been described (21), while immunogenicity of phages generally does not represent a safety risk for patients (19). Coherently with these findings, we demonstrated that the combined treatment is able to downregulate the proinflammatory TNF-α (**Figure 2A**) and Il-1β (**Figure 2B**) cytokine production, while at same time upregulating anti-inflammatory cytokine TGF-β (**Figure 2C**) levels, making it a suitable approach for chronic KPC-KP infection treatment. Additionally, φBO1E alone does not induce changes in uninfected dTHP-1 cell cytokine production profiles, confirming its low immunogenicity.

The results reported herein show that this strategy may improve the current therapeutic regimens to help in preserving the few effective drugs (e.g., colistin and newer beta-lactam/beta-lactamase inhibitor combinations) still available against such pathogens (22). The lack of *in vivo* results and the use of a single bacteriophage strain represent an important limitation to the prompt clinical applicability of our therapeutic strategy. Indeed, preclinical studies are mandatory to solve several safety and efficacy concerns such as (i) phage effect on body tissues or nontarget microbiota, (ii) host immune system phage clearance and degradation, and (iii) the emergence of phage-resistant *K. pneumoniae* strains (19, 23, 24). In this context, our study was designed to be the proof of concept aimed at providing the basis for the necessary improvements required for subsequent preclinical studies, such as the use of PDTs based on phage cocktails or phage/antibiotic combinations. However, it has to be mentioned that φBO1E targets the capsular polysaccharide of KPC-KP of the clade II of ST258 and in *in vivo* model of KKBO-1-infected *Galleria mellonella*, during phage treatment, capsular-deficient *K. pneumoniae* clones can be selected and are characterized by a lower pathogenicity. This evidence suggests that in the context of the interplay among phage, pathogen, and host, the emergence of phage-resistant bacteria may, in this specific case, also be beneficial for the host (25).

Overall, our data support the potential of the combined host and phage therapy as an attractive complement or alternative tool to antibiotic treatment against MDR infections and shed light on an innovative combined approach, targeting at the same time intracellular and extracellular pathogens, improving the clinical management of patients, and limiting the diffusion, the increase, and the spread of MDR pathogen strains.

## Data Availability Statement

The raw data supporting the conclusions of this article will be made available by the authors, without undue reservation.

## Author Contributions

MCT, MMDA, and MF contributed to the conception and design of the study. NP, TO, LHDA, and FDS, contributed to data acquisition. NP, TO, MMDA, and MF participated in data analysis and manuscript writing. All authors read and approved the final manuscript.

## Funding

The research was supported by the Italian Cystic Fibrosis Research Foundation, FFC #21/2019.

## Conflict of Interest

The authors declare that the research was conducted in the absence of any commercial or financial relationships that could be construed as a potential conflict of interest.

## Publisher’s Note

All claims expressed in this article are solely those of the authors and do not necessarily represent those of their affiliated organizations, or those of the publisher, the editors and the reviewers. Any product that may be evaluated in this article, or claim that may be made by its manufacturer, is not guaranteed or endorsed by the publisher.
