# Supplementary material for: Fighting MDR-Klebsiella pneumoniae Infections by a Combined Host- and Pathogen-Directed Therapeutic Approach
Source: Front Immunol. 2022 Feb 14;13:835417. doi: 10.3389/fimmu.2022.835417 (PMC8884248; doi:10.3389/fimmu.2022.835417)
Supplement: Supplementary file 1 [file DataSheet_1.pdf]

Supplementary Material

Fighting MDR- *Klebsiella pneumoniae* infections by a combined host- and pathogen- directed therapeutic approach.

Poerio N.[1a], Olimpieri T.[1a], Henrici De Angelis L.[1,2], De Santis F.[1], Thaller M.C.[1], D'Andrea M.M.[1], Fraziano M[1*].

[1]Department of Biology, University of Rome "Tor Vergata", Rome, Italy

[2] Department of Medical Biotechnologies, University of Siena, Siena, Italy.

[a] *contributed equally to the work*

* Corresponding author:

Maurizio Fraziano: fraziano@bio.uniroma2.it (MF)

Keywords: Liposomes, Phosphatidylinositol 5-phospate, host-directed therapy, bacteriophages, phage therapy, MDR, *Klebsiella pneumoniae*

Method

Cell viability assay. dTHP-1 cells (2× 10^5 per well) were stimulated with ABL/PI5P and/or φBO1E. After 18 hours, cell viability was monitored by the MTT Cell Proliferation Assay Kit (Molecular Probe). Briefly, culture media was removed and replaced with labelling culture media (RPMI 1640 without phenol red, fully supplemented) The MTT assay is based on the cleavage of the yellow tetrazolium salt MTT (3-(4,5-Dimethylthiazol-2-yl)-2,5-diphenyltetrazolium bromide) to purple formazan crystals in metabolically active cells. The formazan is then solubilized using dimethyl sulfoxide (DMSO) 0,01 M, and the concentration determined by optical density at 540 nm. The assay is sensitive with the colorimetric signal proportional to the viable cell number. As negative control, all cell types were treated with 0.1% saponin at 37°C for 30 min. Data are shown as means ± SD of % of cell viability of triplicate cultures. % Cell viability = 100 x Experimental OD_{540nm} / Positive Control OD_{540nm}.

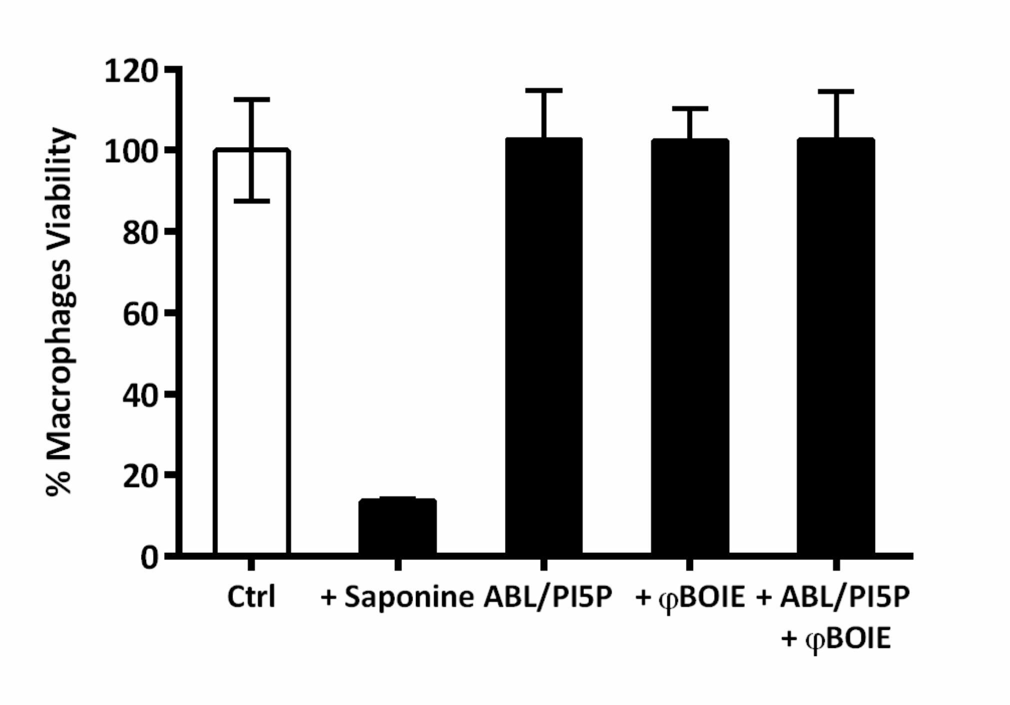

S1. ABL/PI5P and/or φBO1E treatment does not affect human macrophages viability

dTHP1 cells (10^6cells/ml), were stimulated with ABL/PI5P and/or and φBO1E for 18 hours and then cell viability was monitored by the MTT assay. The data are shown as mean ± standard deviation of % of macrophages viability of triplicate cultures and are representative of 3 different independent experiments.
